# Behavioral laterality of the brain: support for the binary construct of hemisity

**DOI:** 10.3389/fpsyg.2013.00683

**Published:** 2013-10-01

**Authors:** Bruce E. Morton

**Affiliations:** John A Burns School of Medicine, University of HawaiiHonolulu, HI, USA

**Keywords:** asymmetry, anterior cingulate cortex, cognition, right vs. left brain orientation, sex differences

## Abstract

Three terms define brain behavioral laterality: hemispheric dominance identifies the cerebral hemisphere producing one's first language. Hemispheric asymmetry locates the brain side of non-language skills. A third term is needed to describe a person's binary thinking, learning, and behaving styles. Since the 1950s split-brain studies, evidence has accumulated that individuals with right or left brain behavioral orientations (RPs or LPs) exist. Originally, hemisphericity sought, but failed, to confirm the existence of such individual differences, due to its assertion that each individual lay somewhere on a gradient between competing left and right brain extremes. Recently, hemisity, a more accurate behavioral laterality context, has emerged. It posits that one's behavioral laterality is binary: i.e., inherently either right or left brain-oriented. This insight enabled the quantitative determination of right or left behavioral laterality of thousands of subjects. MRI scans of right and left brain-oriented groups revealed two neuroanatomical differences. The first was an asymmetry of an executive element in the anterior cingulate cortex (ACC). This provided hemisity both a rationale and a primary standard. RPs and LPs gave opposite answers to many behavioral preference “either-or,” forced choice questions. This showed that several sex vs. hemisity traits are being conflated by society. Such was supported by the second neuroanatomical difference between the hemisity subtypes, that RPs of either sex had up to three times larger corpus callosi than LPs. Individuals of the same hemisity but opposite sex had more personality traits in common than those of the same sex but different hemisity. Although hemisity subtypes were equally represented in the general population, the process of higher education and career choice caused substantial hemisity sorting among the professions. Hemisity appears to be a valid and promising area for quantitative research of behavioral laterality.

## Introduction

Awareness of laterality of brain function is at least as old as written history. For example, Diocles of Carystus in the 4th century BC insightfully wrote:

There are two brains in the head, one which gives understanding, and another which provides sense-perception. That is to say, the one which is lying on the right side is the one that perceives: with the left one, however we understand. (Lockhorst, 1985)

However, Marc Dax was the first in modern times to observe a difference in function between the hemispheres. In 1836 he noticed that victims of injury to the left hemisphere (LH) but not to the right hemisphere (RH) could not speak (Dax, 1865). Paul Broca extended this work by additionally noting that often the dominant hand was contralateral to the language hemisphere (Broca, 1865).

### Hemispheric dominance vs. hemispheric asymmetry

For the following century, the term “hemispheric dominance” was only used to refer to language laterality of the brain. Then, a large study by Weisenberg and McBride (1935) demonstrated RH excellence in visuospatial skills. This called for the invention of a second term, “hemispheric asymmetry,” to describe the many more-recently discovered non-language differences in cerebral structure and function, most notably those revealed in “split-brain” subjects. These individuals had been created by treatment for intractable epilepsy by cutting the corpus callosum, the main cerebral connection between the hemispheres, thus limiting the spread of seizures from one side to the other (Gazzaniga et al., 1962, 1967; Sperry, 1982; Gazzaniga, 2000).

Based upon the surprisingly different responses obtained from each of these isolated hemispheres within split-brain subjects (Gazzaniga et al., 1962, 1967; Geschwind et al., 1995; Gazzaniga, 2000), it was early proposed by investigators that the right and left cerebral hemispheres are characterized by inbuilt, qualitatively different and mutually antagonistic modes of data processing, separated from interference by the major longitudinal fissure of the brain (Levy, 1969; Sperry, 1982). In this model, the LH specialized in top-down, deductive, cognitive dissection of local detail. In contrast, the RH produces a bottom-up, inductive, perceptual synthesis of global structure (Sperry, 1982; Schiffer, 1996; Gazzaniga, 2000). This functional asymmetry context has been reinforced by known laterality differences between them. That is, there are striking differences in input to each hemisphere, differences in internal neuronal-columnar architecture, and differences in hemispheric output (Kosslyn et al., 1989, 1992; Schuz and Preissl, 1996; Hutsler and Galuske, 2003; Jager and Postma, 2003; Stephan et al., 2006) that support a local wiring on the left vs. global wiring motif on the right.

Congruent with the above local-global view is a large body of detailed evidence that the left cerebral hemisphere in most right-handed individuals manifests facilities for language (Broca, 1865), has an orientation for local detail (Robertson and Lamb, 1991), has object abstraction-identification abilities (Kosslyn, 1987), and appears to possess a hypothesis-generating, event “Interpreter” (Gazzaniga, 1989, 2000; Wolford et al., 2000). In contrast, the RH has been demonstrated to excel in global analysis (Robertson and Lamb, 1991; Proverbio et al., 1994), object localization (Kosslyn et al., 1989), facial recognition (Milner, 1968), and spatial construction (Sperry, 1968).

Among the about 90% of humans who are right-handed (Coren, 1992), language is located in the LH in about 96% of them (Knecht et al., 2000). Of the remaining about 10% of left handed individuals, some 73% of these also have language in their left cerebrum (Knecht et al., 2000). Thus, by simple arithmetic it follows that that the LH houses language ability in about 93.7% of us.

### Hemisphericity

It is of interest here that within this huge group of right handed, LH dominant speakers, the existence of two major human sub-populations has repeatedly been inferred (Sperry, 1968, 1982; Bogen, 1969; Levy, 1969; Bradshaw and Nettleton, 1981; Kosslyn, 1987; Robertson and Lamb, 1991; Davidson, 1992; Schiffer, 1996; Springer and Deutsch, 1998), whose characteristic thinking and behavior styles differ in a manner that appeared to mirror the putative properties of the asymmetric hemispheres. That is, in some right-handed, LH languaged individuals, putative LH traits seemed to be ascendant, to produce a “Left brain-oriented” thinking and behavioral style (Fink et al., 1996; Springer and Deutsch, 1998). Such left brain-oriented persons are currently summarized as top-down, detail-oriented, deductive, “splitters.” Yet, in another equally large group of right-handed LH languaged persons, RH traits are thought to be more prominent, resulting in a contrasting “Right brain-oriented” style (Davidson and Hugdahl, 1995; Schiffer, 1996), currently viewed as bottom-up, global, inductive, “lumpers.”

Thus, the original permanent assignment of the terms “hemispheric dominance” to language laterality, and “hemispheric asymmetry” to non-motor lateralities ultimately forced the creation of a third asymmetry term, that of “Hemisphericity” (Bogen, 1969; Bogen et al., 1972) in order to describe this third phenomenon, behavioral laterality style. This term was needed in order to refer to the differences in left and right brain thinking and behavioral properties within the two groups of individuals with language dominance and non-language asymmetry commonalities.

Why should hemisphericity exist? Upon what mechanism might these two thinking and behavioral styles of hemisphericity depend? Early studies of this phenomenon were doomed by misconception that hemisphericity was the result of hemispheric competition (Corbalis, 1980; Bradshaw and Nettleton, 1981; Beaumont et al., 1984). This resulted in hundreds of conflicting reports. For example, many studies found the presence of frontal EEG alpha asymmetries related to emotional states [reviews by Davidson (1984a,b, 1988)]. State-independent or trait-related individual differences in EEG asymmetries related to affective valence have also been described, [reviews by Davidson and Tomarken (1989); Davidson (1992)].

Similarly, another commonly employed measure of hemisphericity has been the predominant direction of conjugate lateral eye movements (CLEMs) in response to questions requiring reflective thought. CLEMs have been proposed as a measure of relative hemispheric activation, greater on the side contralateral to the direction of eye movement (Kinsbourne, 1972, 1974; Bakan and Strayer, 1973; Gur, 1975). Both EEG and CLEM lateralities seem related to hemispheric emotional asymmetry, but do not appear to be valid predictors of differences within normal behavior (Beaumont et al., 1984; Reine, 1991).

Further, within the formal definition of hemisphericity, attempts to keep the discipline of psychology scientific demanded each person to be located somewhere on a gradient between putative left and RH behavioral extremes. Because most subjects hesitate to mark extremes (Dawes, 2008), this impeded the development of usable quantitative methods needed to determine individual hemisphericity. After thousands of conflicting reports, the field of hemisphericity collapsed in the 1980s, primarily due to these foundational misunderstandings and this unhelpful definition, (Beaumont et al., 1984; Efron, 1990; Fink et al., 1996; Schiffer, 1996; Ornstein, 1997; Springer and Deutsch, 1998). Hemisphericity has since been called a neuromyth that was debunked in the scientific literature 25 years ago (Corbalis, 1980; Lindell and Kidd, 2011). As a result, publications have plummeted so that over the last 20 years the term hemisphericity has appeared in the title of only seven publications listed in Medline, aside from those of this author. In contrast, other aspects of brain laterality, such as handedness or language dominance, have hundreds of publications over the same period. Recently, a further nail in the coffin of hemisphericity has been supplied by the observation that no individual or group differences in lateral brain activity could be seen by functional magnetic resonance imaging (fMRI) (Nielsen et al., 2013).

### Hemisity

A quarter of a century after the “death” of hemisphericity and of the consequent loss of a valid and needed term to describe the brain behavioral laterality of individuals, a new more accurate approach to behavioral laterality term was created, called “Hemisity,” (Morton and Rafto, 2010). Unlike hemisphericity, hemisity is binary; thus matching the other two binary descriptors of brain behavioral laterality: hemispheric dominance and asymmetry (Table 1). In this new context, an individual is inherently, unavoidably, and irreversibly either left, or right brain-orientated in thinking and behavioral style, and in a manner quite unrelated to hemispheric competition. Thus, hemisity has restored a valid descriptor for the above mentioned essential third element necessary to describe brain laterality. The author entered the field in 2001 with this binary distinction, but initially published his results under the term of hemisphericity.

**Table 1:** **Three essential cerebral hemisphere laterality terms**.

*Hemispheric Dominance:* A valid term that refers to which cerebral hemisphere houses first language production skills.
*Hemispheric Asymmetry:* A valid term that refers to which hemisphere produces the various non-language skills, such as facial recognition, emotion recognition, emotion production.
*Hemisphericity*: An obsolete term that tried to describe an individual's characteristic learning and behavioral style as being located somewhere on a gradient between right and left brain extremes.
*Hemisity:* A term replacing hemisity that refers to which hemisphere inherently contains an individual's unilateral executive element, the source of their characteristic learning/behavioral style. Thus, each person is inherently either left or right brain-oriented. Adding sex, the other binary identifier, produces the four major hemisity subtypes: RM, RF, LM, and LF. This situation requires rethinking of sexual characteristics, which are presently being conflated with hemisity subtype characteristics.

## Biophysical and questionnaire measures of hemisity

In contrast to analog hemisphericity, the binary “hemisphericity” (hemisity) concept was more in alignment with the qualitatively different and mutually antagonistic modes of data processing of the opposite cerebral hemispheres, and certainly was much easier to quantify. Numerous “hemisphericity” reports were published (Morton, 2001, 2002, 2003a,b,c,d; Morton and Rafto, 2006). This series was continued by publication of additional “hemisity” reports (Morton and Rafto, 2010; Morton, 2012; Morton et al., 2014).

First, four independent biophysical methods were devised to separate right and left brain- oriented persons (RPs and LPs). Each of these showed a remarkable consistency in dividing large groups of individual into nearly the same groups of LPs and RPs. Based upon the identity of these hemisity subgroups, ultimately four “either-or” forced choice preference type questionnaires were created whose applications also divided a large starting group into the same RP and LP hemisity subgroups. These biophysical and derivative questionnaire methods are briefly described next.

### Dichotic deafness task

Morton (2001) reported that normal subjects could be segregated into two groups on the basis of the Dichotic Deafness Test, a dichotic listening task involving the simultaneous presentation of non-matching pairs of consonant-vowel syllables (CV). “Dichotically hearing” subjects reported more than 40% of the syllables presented to their minor (left) ear compared to their major (right) ear, while “dichotically deaf” subjects reported less than 40% of the CV syllables presented to their minor ear. Forty percent was an arbitrary bootstrapping value empirically found to provide optimal separation of the two groups. Morton (2002) found that dichotically hearing subjects affirmed predominantly right hemisphericity items on Zenhausern's Preference Questionnaire (Zenhausern, 1978), while dichotically deaf subject showed a left brain orientation.

### Polarity questionnaire

Morton (2002) described the development of a new hemisity questionnaire, The Polarity Questionnaire, the items of which were chosen for their ability to differentiate groups of subjects divided on a priori grounds into left and right hemisity groups. Grouping into dichotically hearing (right brained) and dichotically deaf (left brained) groups of subjects, defined by the Dichotic Deafness Test, showed a very strong correlation with the Polarity Questionnaire (*r* = 0.51, *p* < 0.001). This correlation was twice the magnitude of the correlation between the Dichotic Deafness Test and Zenhausern's Preference Questionnaire (Zenhausern, 1978). Only 30% of the Zenhausern's Preference Questionnaire items, vs. 90% of the Polarity Questionnaire items, were significantly correlated with Dichotic Deafness Test grouping. A low correlation between the Polarity Questionnaire and Zenhausern's Preference Questionnaire was also noted by McElroy et al. (2012) andby Morton (2012).

### Mirror tracing task

Morton (2003a) had right handed subjects trace the outline of a five-pointed star as quickly as possible with either hand, using only a mirror to guide manual circumscription. Faster mirror tracing with one hand was regarded as an indication of preference for the use of the contralateral hemisphere. In the total sample of subjects, mirror tracing asymmetry was not significantly correlated with the Dichotic Deafness Test, Zenhausern's Preference Questionnaire, or the Polarity Questionnaire. However, when subjects identified as having left brain affect by use of the Affective Laterality Test (Schiffer, 1997) were removed, robust correlations between mirror tracing asymmetry and the other three hemisity measures were observed. In the Affective Laterality Test, the hemisphere which is more responsive to emotionally-evocative pictures is determined. This is done by having subjects view pictures while wearing goggles which restrict vision to the periphery (viewing with the nasal portion of the retina) by occluding the inner two thirds of each lens, thus allowing viewing by only one hemifield of one eye at a time. Subjects are asked to judge which viewing eye was associated with larger initial emotional responses to the pictures. The validity of this approach was confirmed (Schiffer et al., 2007). When the hemisity outcomes on the mirror tracing test were reversed or “phase corrected” for subjects with left brain affect (greater emotional responses to pictures viewed with the nasal portion of the right eye) and these data were included in the analysis, even larger correlations with the other three hemisity measures were evident (Morton, 2003a).

### Best hand task

Extending a line bisection instrument of Schenkenberg et al. (1980), Morton (2003b) had subjects draw a line through the estimated midpoint of a set of lines of varying lengths with each hand. Midpoint estimates for each hand of an individual showed excellent repeatability and stability. When the midpoint estimates of opposite hands were compared, characteristic and often large individual differences between the accuracy of each hands to bisect the lines were observed.

Of the 412 subjects studied, 75% fell into two of the four line-bisection response categories based on the more accurate hand (*r* or *l*) and whether it crossed over the other hand to mark (c) or it did not cross over, but marked on the same (s) side as the other hand. That is, the rs category = 45% and *lc* = 30%. Most of rs-category subjects uncorrected for handedness or left-handed writing grasp were classified as left brained by the Polarity Questionnaire. Conversely, most of the subjects in the *lc*-category were classified as right brained by the Polarity Questionnaire.

For the two smaller categories, the results were somewhat more complicated. Of the 10% of the total sample who fell into the rc-category, the males were right brained (8%), while the females were left brained (2%). Of the 15% of the total sample who fell into the ls-category, those with right brain affect on the Affective Laterality Test were right brained, as determined by the Polarity Questionnaire (10%), whereas those with left brain affect had left hemisity (5%). Thus, hemisity as determined by phase-corrected line-bisection results was also strongly associated with hemisity, as determined by phase-corrected mirror tracing results, the Dichotic Deafness Test, and Zenhausern's Preference Questionnaire.

### Asymmetry questionnaire

Morton (2003c) developed another questionnaire measure of hemisity, the Asymmetry Questionnaire, which consists of 15 paired statements. Within each pair, one statement exemplified a left brained characteristic while the other reflected a right brained characteristic. The Asymmetry Questionnaire was found to have strong and significant correlations with two other hemisity questionnaires, the Polarity Questionnaire and Zenhausern's Preference Questionnaire, as well as three biophysical hemisity measures, the Dichotic Deafness Test, phase-corrected mirror tracing, and phase-corrected Best Hand Test.

### Binary questionnaire and hemisity questionnaire

Recently the Binary Questionnaire and the Hemisity Questionnaires have also been developed and utilized (Morton, 2012). As shown in Table 2, these were of comparable quality to the Polarity and Asymmetry Questionnaires. As may be seen, all four of these questionnaires were superior to the earlier hemisphericity standard, the Zenhauser's Preference Questionnaire (1978).

**Table 2:** **Overall correlations and reliability of preference questionnaire scores with predetermined subject hemisity subtype**.

**Preference questionnaires (fast, easy) vs. biophysical methods (slow, difficult)**	***r* (Pearsons)**	***p***	***n***	**% yield**	**alpha Cron-bach's**
**CORRELATIONS OF MRI PRE-ASSIGNED HEMISITY SUBTYPES WITH**					
Zenhausern's preference quest-naire	0.24	0.008	119	35^*^	0.37
Polarity questionnaire	0.57	0.000	132	82	0.57
Asymmetry questionnaire	0.48	0.000	111	60	0.64
Binary questionnaire	0.43	0.000	112	30	0.66
Hemisity questionnaire	0.53	0.000	79	48	0.65
Best hand test (R − L)	0.37	0.000	143		
Mirror tracing test (R/L)	0.50	0.000	116		
Dichotic deafness test (R − L/R + L)	0.34	0.000	109		
vgACC laterality determined by MRI	0.93	0.000	149		

* =% yield refers to the percentage of questionnaire statements that were significantly associated with subject neuroanatomical hemisity. Pre-assigned hemisity subtype = direction of asymmetry of the ventral gyrus of the anterior cingulate cortex.

## MRI studies of neuroanatomical differences between RPs and LPs

The above new methods enabled the accurate characterization the hemisity subtype of hundreds of subjects (Morton, 2003d). This enabled MRI studies to be carried out seeking brain structural differences between LPs and RPs. Two neuroanatomical differences were found. The first was the observation that the corpus callosum midline cross sectional area of RPs was up to three times larger than that of the LPs (Morton and Rafto, 2006). The implications of this discovery will be discussed later. Second, it was observed that in 146 of 149 cases (98%) the subject's bilateral anterior cingulate cortex (ACC) in Areas 24 and 24′ was up to 50% larger on the right side for RPs, while for the LPs it was up to 50% larger on the left (Morton and Rafto, 2010), Figure 1. This result motivated the transformation of this 3 min MRI procedure into the primary standard for the determination of individual hemisity subtype, as follows:

**Figure 1:**
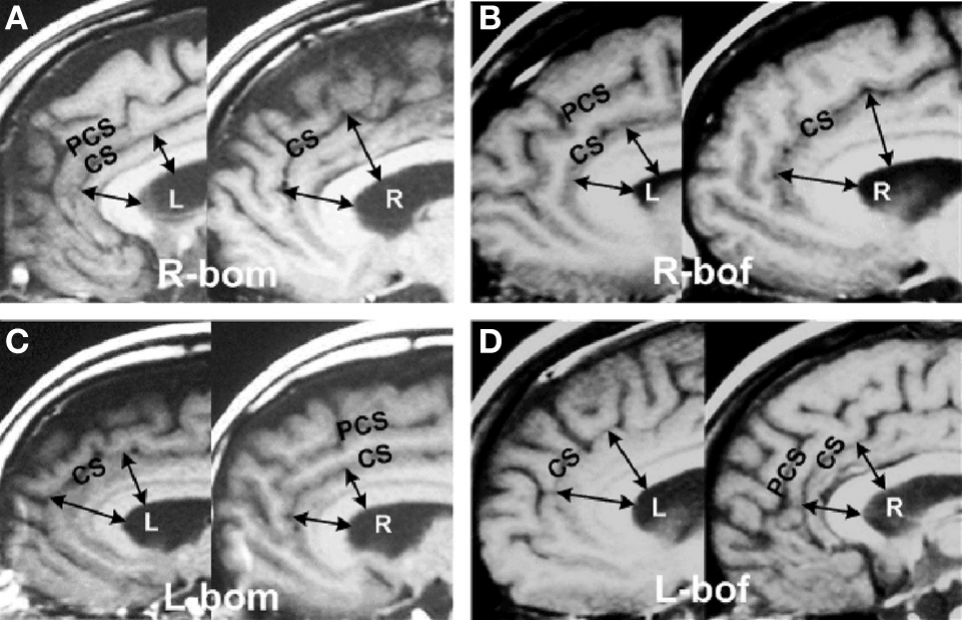
**Asymmetries in the anterior cingulate cortex**. Example of MRI sagittal images taken from 149 hemisity-calibrated subjects. **(A)** Right brain-oriented male (R-bom, RM). **(B)** Right brain-oriented female (R-bof, RF). **(C)** Left brain-oriented male (L-bom, LM). **(D)** Left brain-oriented female (L-bof, LF). Pairs of arrows reaching from the lower surface of the central white corpus callosum (CC) to the cingulate sulcus (CS) illustrate four measurements made for each subject. CC thickness was the same on images from either side. PCS refers to the paracingulate sulcus. Note that the arrow lengths are longer on the right side for RPs and left side for LPs. From Morton and Rafto (2010).

### MRI assessment of hemisity (primary standard)

MRI assessments (Morton and Rafto, 2010) were obtained employing a General Electric Signa 1.5 Tesla MRI instrument. A midsagittal plane setup calibration protocol was run for 3 min to image 5 mm thick slices from the midline plane and two adjoining sagittal planes 6 mm on either side. Whole-head photographic images were prepared from these three planes. These three exposures were printed on a single film sheet for each subject. This procedure enabled both cortical walls on either side of the midline fissure to be visualized and measured, thus allowing sub-element lateralities of the ACC to be evaluated directly from the film. At two ACC sites on each side of the brain, one in Area 24 and the other at Area 24′ (Vogt et al., 1995), estimations of the relative thickness of the ventral gyri (vgACC) there were made. This abbreviation and these four ACC locations within Areas 24 and 24′ are not to be confused with the more frontal ventral region of the perigenual ACC. The vgACC locations where these relative thickness estimations were made are illustrated by the arrows in Figure 1.

Two lines were extended outward perpendicularly from the inner edge of the CC, ending in one case at a more frontal point in Area 24 and in the other at a more dorsal point in Area 24′. Both points were in the plane of the cingulate sulcus and arbitrarily selected, based upon the sites in the region giving the largest vgACC thickness for each brain side involved. The average of these two lateral relative thickness estimates from the vgACC of each side were then used to determine upon which side of each subject's brain the vgACC was thicker. This can be recognized by noting that the arrows are longer on the RH for RPs and on the left for LPs.

### Calibration of earlier hemisity methods against the MRI primary standard

Asymmetry of the ventral gyri of the ACC was significantly correlated with hemisity as determined by the Asymmetry Questionnaire (Morton, 2003c), the Polarity Questionnaire and Zenhausern's Preference Questionnaire (Zenhausern, 1978; Morton, 2002), the Dichotic Deafness Test (Morton, 2001, 2002), the Best Hand Test (Morton, 2003b), the Phased Mirror Tracing Test (Morton, 2003a), as well as two new hemisity questionnaires, the Binary Questionnaire and the Hemisity Questionnaire (Morton, 2012). The categorical associations of each of these methods of determining hemisity with each other and with asymmetry of the vgACC were highly significant (Morton and Rafto, 2010). The correlations among continuous measures of asymmetry derived from each of these methods were also significant. All nine hemisity measures had high loadings on the first factor, suggesting an underlying dimension of hemisity accounting for the relationships among these nine measures. The correlations between these hemisity instruments may be seen in Table 2.

That the anatomical primary standard for hemisity was found to validate the previous secondary instruments developed to assess hemisity was gratifying because some of them were based upon possibly questionable assumptions. For example, in the Dichotic Deafness Test (Morton, 2001), it was necessary to make arbitrary decisions as to where to draw cutoff lines that defined dichotic deafness. In the Phased Mirror Tracing Method (Morton, 2003a) it was necessary to assess the subjects as to which was the more emotional side of their brain. This assessment was based upon the examiner's interpretation of the subjective judgment of the subject in response to peripheral presentation of pictures containing emotion-invoking content. In the Best Hand Task (Morton, 2003b), a certain segment of the population required redefinition of handedness and the interpretation of the sometimes-difficult assessment of pen grasp hand posture. It is paradoxical that it was necessary to develop these secondary methods first in order to calibrate the hemisity of a sufficiently large group of subjects even to begin to search for and recognize actual brain structural differences between left and right brain-oriented individuals.

However, since the previous hemisity procedures were well correlated with the primary anatomical standard, it would appear reasonable they could continue to be used in combination as secondary standards. When five of these six were used the combined outcome for the 149 subjects was 146/149 (98%) correct for hemisity subtype identity. For the 111 subjects assessed by all six secondary methods, the accuracy rose to 99%. Yet, no single secondary method can be used to absolutely identify subject hemisity, each being correct only about 80% of the time. It would appear that, the combined use of at least three or four of the five most accurate questionnaires of Table 2, would allow for rapid, fairly accurate measurement of the hemisity of individuals. In sufficiently large populations, this can be reduced to two hemisity questionnaires, as described later.

## Neuroanatomical basis of hemisity

Coincidentally in terms of the hemisity MRI findings of ACC laterality, much evidence supports the ACC being a major structural element of the brain's executive system. Remarkably, this cortical element of the ancient limbic brain region (Roxo et al., 2011), including interconnecting integrative loops (Alexander et al., 1986) between prefrontal, striatal, thalamic, and other limbic areas (Bonelli and Cummings, 2007) has repeatedly been shown to be involved in executive type activities. These include: decision making (Kennerly et al., 2006), error detection, conflict monitoring, stimulus-response mapping, familiarity, and orienting (Wang et al., 2005), response to pain and production of emotion: (Vogt, 2005), verbal and non-verbal executive tasks activity (Fornito et al., 2004), conflict monitoring and adjustments in control (Kerns et al., 2004), rapid processing of gains and losses (Gehring and Willoughby, 2002), interfacing between motor control, drive, and cognition (Paus, 2001), episodic memory retrieval (Herrmann et al., 2001) and the initiation and motivation of goal directed behavior (Devinsky et al., 1995).

Some ACC activities appear directly relevant to hemisity differences in behavioral styles. These include its participation in temperament (Whittle et al., 2008), reward and social learning (Behrens et al., 2008), expectancy and social rejection, Somerville et al. (2006), self-reflection (Johnson et al., 2006), personality (Pujol et al., 2002), will and addiction (Peoples, 2002). Even though psychoanalytic concepts were originally not intended to correspond to neuroanatomical structures, it can be noted that the ACC seems to mediate a number of different cognitive functions formerly subsumed under Freud's central element of control, the Ego. It certainly has the resources to implement the many behavioral differences between hemisity subtypes.

What is fascinating in terms of the hemisity story, is that not only does the ACC house a major brain executive element, but also that its two sides, separated by the cerebral midline fissure, are highly asymmetric. There are at least 10 reports of ACC structural asymmetries, especially in Areas 24, and 24′ which varied in an individually idiosyncratic manner, (Vogt et al., 1995; Paus et al., 1996a,b; Hutsler et al., 1998; Ide et al., 1999; Yucel et al., 2001; Pujol et al., 2002; Fornito et al., 2006, 2008; Huster et al., 2007; Palomero-Gallagher et al., 2008). Many of these reports mentioned efforts to identify behavioral consequences of these identified asymmetries, interestingly including their possible relationship to executive function, e.g., Pujol et al. (2002). However, these efforts lacked the unifying concept of hemisity.

Might this laterality of the ACC executive element provide a direct link to a subject's hemisity, thus supporting the observed relationship between the two? Indeed, it is here asserted that the discovery of the congruity of the larger side of the ACC with hemisity subtype has actually provided the missing mechanism to account for the existence of hemisity and for the differences between LPs and RPs. Further, such an “either-or” laterality context is consistent with the logic that there can be only one “Bottom-line,” “The buck stops here” executive element in any successful institutional organization, including the mammalian brain, which is completely bilateral, except for the pineal gland. Although, Descartes (1637) was logically compelled to assert this endocrine organ to be the executive “Seat of the Soul,” now, it rather appears that the executive system must be unilateral. That is, hemisity must result because an executive element, embedded in the local specialized (top-down, important details) environment of the LH, will inevitably have a different perspective than one imbedded within that of the right (bottom-up, global perspective).

Thus, the existence of major asymmetries in the ACC supports the hypothesis of the possible existence of a unilateral executive element. This idea is not new. When he learned that the bilateral ACC was the probable site of the executive system, Crick (1994) was led rhetorically to ask: “Could there be two centers of the Will?” (Sejnowski, 2004). In a “Postscript on the Will” within his book “The Astonishing Hypothesis,” (1994), Crick states that he and Antonio Damasio arrived at the same negative answer to this question by noting about the ACC that the “region on one side projects strongly to the corpus striatum (an important part of the motor system) on both sides of the brain, which is what you might expect from a single Will.” Parenthetically, neither their use of the term Will, nor the use of the term Executive System here were intended to invoke the idea of a decisional homunculus, but rather of a preconscious early response system (Libet, 1982) continually acting to optimize the survival of the organism.

## Behavioral differences between right and left hemisity subtypes

With the ability to accurately determine the right or left brain individual hemisity subtype identity in hand, it became possible to answer some pressing questions: do these biophysically identified right and left hemisity subtype individuals differ significantly in their behavioral preferences? And if so, specifically how? Morton (2012) studied the behavioral responses of 150 subjects whose hemisity had previously been calibrated by MRI. He used five MRI-calibrated preference questionnaires, two of which were new. Right and left brain-oriented subjects selected opposite answers (*p* > 0.05) for 47 of 107 “either-or,” forced choice type preference questionnaire items. Removing overlaps resulted in 30 hemisity subtype preference differences (Table 3). These differences could be subdivided into five areas: (1) in logical orientation, (2) in type of consciousness, (3) in fear level and sensitivity, (4) in social-professional orientation, and (5) in pair bonding-spousal dominance style.

**Table 3:** **Thirty binary behavioral correlates of hemisity**.

**Left brain-oriented persons**	**Right brain-oriented persons**
**LOGICAL ORIENTATION**	
Analytical (stays within the limits of the data)	Sees the big picture (projects beyond data, predicts)
Uses logic to convert objects to literal concepts	Imagines, converts concepts to contexts or metaphors
Decisions based on objective facts	Decisions based on feelings, intuition
Uses a serious approach to solving problems	Use a playful approach to solving problems
Prefers to maintain and use good old solutions	Would rather find better new solutions
**TYPE OF CONSCIOUSNESS**	
Daydreams are not vivid	Has vivid daydreams
Doesn't often remember dreams	Remembers dreams often
Thinking often consists of words	Thinking often consists of mental pictures or images
Can easily concentrate on many things at once	Tends to concentrate on one thing in depth at a time
Comfortable and productive with chaos	Slowed by disorder and disorganization
Often thinking tends to ignore surroundings	Observant and in touch with surroundings
Often an early morning person	Often a late night person
**FEAR LEVEL AND SENSITIVITY**	
Conservative, cautious	Innovative, bold
Sensitive in relating to others	Intense in relating to others
Tend to avoid talking about emotional feelings	Often talks about own and others feelings of emotion
Suppresses emotions as overwhelming	Seeks to experience and express emotions more deeply
Would self-medicate with depressants	Would self-medicate with stimulants
**SOCIAL AND PROFESSIONAL ORIENTATION**	
Does not read other people's mind very well	Good at knowing what others are thinking
Thinks-listens quietly, keeps talk to minimum	Thinks-listens interactively, talks a lot
Independent, hidden, private, and indirect	Interdependent, open, public, and direct
Does not praise others nor work for praise	Praises others and works for praise
Avoids seeking evaluation by others	Seeks frank feedback from others
Usually tries to avoid taking the blame	Tends to take the blame, blames self, or apologizes
**PAIR-BONDING AND SPOUSAL DOMINANCE STYLE**	
Tolerates mate defiance in private	Finds it difficult to tolerate mate defiance in private
After an upset with spouse, needs to be alone	After upset with spouse, needs closeness and to talk
Needs little physical contact with mate	Needs a lot of physical contact with mate
Tends not to be very romantic or sentimental	Tends to be very romantic and sentimental
Prefers monthly large reassurances of love	Likes daily small assurances of mate's love
Often feels mate talks too much	Feels my mate doesn't talk or listen enough
Lenient parent, kids tend to defy	Strict, kids obey and work for approval

The following is an interpretation of 30 hemisity differences found: regarding *Logical Orientation*, LPs tended to be top-down, detail oriented, and deductive vs. RPs who were more bottom-up, big picture, and inductive. Regarding *Type of Consciousness*, LPs tended to be more verbal, dependent upon abstract reasoning, and oriented to find differences between objects vs. RPs who where more visual, dependent upon concrete reasoning, and able to find commonalties between objects. As to *Fear Level and Sensitivity*, LPs were more sensitive, taciturn, emotion-avoiding and defensive (implying a thinner barrier to fear-invoking subconscious material), while RPs were more intense, bold, talkative, emotion-embracing, and invasive. For *Social and Professional Orientation*, LPs were more independent, avoidant, private, and competitive, while the RPs were more orderly, responsible, open, and cooperative. In terms of *Pair Bonding Style and Spousal Dominance*, LPs were the less dominant spouse, who needed separateness, quietness, seeking to avoid emotionality with logic, spouse assisting, and initiator of the details of family endeavors early in the day. In contrast RPs were the more dominant spouse, needing closeness and reassurance of the other's fidelity and support while being intuitive and highly directive, ending the day by reviewing the big picture survival status of the family and making plans for the next day.

It is ironic that many of these behavioral preference differences parallel some, but not all, of the putative differences between the right and left brainers popular in folk hemisphericity (Springer and Deutsch, 1998), such as detailer vs. globalist, analytical vs. synthetic, words vs. images, abstract vs. concrete (L vs. R, here). However, many more differences were revealed, most of which as yet have no recognized brain basis, for example fear vs. confidence, or morning vs. evening, quiet vs. talkative. Perhaps the use of hemisity to identify individuals with those traits may assist in identification of their underlying brain mechanism.

## Corpus callosal size, hemisity, and sexual stereotyping

**Figure 2:**
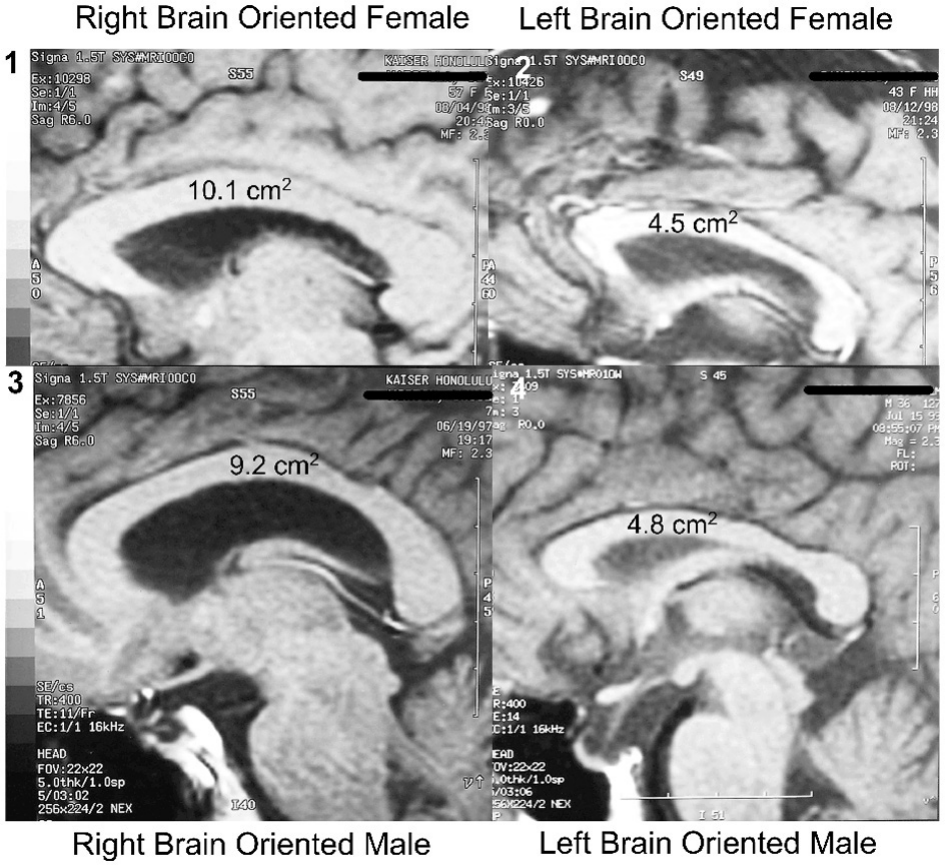
**Hemisity vs. sex: size range of corpus callosal areas**. Largest CCAs of the subject group (*n* = 113): **(1)** Right brain-oriented female, 10.1 cm^2^. **(3)** Right brain-oriented male, CCA 9.2 cm^2^. Smallest CCAs: **(2)** Left brain-oriented female, 4.5 cm^2^. **(4)** Left brain-oriented-male, 4.8 cm^2^. From Morton and Rafto (2006).

As mentioned, the cross-sectional area of the midline of the corpus callosum (CCA) was found to be significantly smaller in LPs than in RPs, and to be unrelated to sex or handedness (Morton and Rafto, 2006). These observations, illustrated in Figure 2, have had several ramifications. To begin with, if the executive element of the anterior cingulate was in the same hemisphere as language, as is the case for most LPs, there would be less need for transcallosal communication than if the executive element was located in the opposite non-language hemisphere. Thus, the CCA in LPs would be predicted to be smaller than in RPs, as observed.

Further, hemisity behavioral outcomes contradict several commonly held beliefs about sex and the brain: first, the hemisity results lay bare the underlying basis of the previous controversy about gender and laterality. The confusion occurred because in all earlier CCA studies, the hemisity of the subjects was unknown. This caused an unwitting confounding of the results for subjects sorted only by sex or handedness with hemisity, a major factor influencing CCA (Morton and Rafto, 2006). This error brings into question the common view that the male brain is more specialized due to its higher laterality (McGlone, 1980). Rather, the CCA data strongly suggest that it is the left brain-oriented individuals of either sex who are more lateralized as a class than males are. Correspondingly, right brain individuals of either sex are less lateralized and more broadly generalized as a class than females are, thus contradicting another sexual stereotype.

Second, these findings appear to end the controversy about which sex has the larger corpus callosum (Luders et al., 2003). There was no significant difference between the two sexes in either their mean CCA, its size range, or in the IQ of the subjects (Morton and Rafto, 2006). Rather, the two largest CCAs of individuals from among our 113 subjects were possessed by a right brained female and by a right brain male (10.1 and 9.2 cm^2^, respectively). Conversely, the two smallest CCAs were 4.8 cm^2^ for a left brained male and 4.5 cm^2^ for a left brained female. All four of these individuals held doctoral degrees and professorial status.

Third, lack of awareness that hemisity contributes to CCA makes it probable that the European studies reporting mean CCAs for males to be larger (Clarke et al., 1989) and American–Australian studies, showing larger female mean CCAs (Holloway et al., 1993) were both correct. Their disagreements could well be based upon regional population differences in hemisity, an important but uninvestigated topic.

Fourth, it is becoming clear that members of either sex with the same hemisity have more behavioral traits in common than do same sex individuals of the opposite hemisity. This is strongly supported by data from the MRI calibrated preference questionnaires (Morton, 2002, 2003c, 2012). Thus, it would appear that several hemisity traits are presently being misidentified as male or female sex traits. That is, men in general do not “hide in their caves of silence” (Tannen, 1990; Gray, 1992). In fact, in contrast to their right brain counterpart, left brain-oriented females are every bit as “private” as left brain-oriented males (Morton, 2002, 2003c, 2012). Similarly, females do not always “rule the roost.” It is the right brain-oriented person who tends to dominate the nuclear family, be they male or female (Morton, 2002, 2003c, 2012). Because of the newness of hemisity and its new behavioral distinctions, sex traits have never been studied together with hemisity traits. Books such as John Gray's “Men are from Mars, Women are from Venus” (1992) appear to fit perfectly for about half the population (~60%), that is, for the RFs and LMs. The other half (~40%) say it is totally alien to them. However, if the pronouns are reversed from “him” to “her” and vice versa in the book, then the other half of the population (RMs and LFs) strongly identify with it (Morton, unpublished). So it appears not to be a description of sexual differences but rather of hemisity differences. Thus, the recognition of the quantifiable existence of hemisity can bring new clarity to human behavior.

## Hemisity distributions and hemisity sorting within populations

Morton (2003d) investigated the distribution of hemisity subtypes within the general population. It was proposed (Morton, 2003d) that in an unsorted population not only would the numbers of male and females be equal, but that the numbers of RPs and LPs would also be similar. It was hypothesized that hemisity sorting in populations would only occur after admission into a school or an organization where entrance was competitive and selective. In the US, this typically first occurs at the university level because in essentially all public elementary, high schools, and even some community colleges, essentially no applicants are excluded and all must complete a similar general core curriculum in order to graduate.

Morton et al. (2014), using the Best Hand Test (Morton, 2003b) and the Polarity Questionnaire (Morton, 2002), measured the hemisity of 1049 public high school upper classmen from Hawaii and Utah. As predicted, in this sample there were similar numbers males (*n* = 522) and females (*n* = 527), and of right (*n* = 526) and left (*n* = 523) brain-oriented individuals. There were reciprocal complementary relationships between right males (RMs, 39%, *n* = 206) and left females (LFs, 40%, *n* = 210), and correspondingly among left males (LMs, 61%, *n* = 316) and right females (RFs, 60%, *n* = 317), thus confirming the non-sorting hypothesis. This suggests that females are slightly enriched in RPs and males are with LPs, and therefore that the average CCA of females should be slightly greater than of males. However, these differences do not appear to obviate the four generalities of the preceding section.

The equalities of hemisity within the general population were lost among 228 competitively selected college freshmen, 57% of whom now showed left hemisity. Students in more specialized upper- division classes (Morton, 2003d) showed an increased range of hemisity distributions, from 35% left brained individuals in a civil engineering seminar to 68% left brained persons in a home economics course.

Even more pronounced hemisity distribution differences were found in university representatives of 17 different professions, ranging from only 21% left brained among astronomers and 33% left brained among architecture professors, to 83% among biochemistry professors and 86% among microbiology professors (Morton, 2003d). Professional librarians (*n* = 15) were predominantly left-brained (73% LPs), while academically trained musicians (*n* = 91) including concert pianists (*n* = 47) were predominantly right-brained (32% LPs) (Morton et al., 2014).

Within professional groups there were differences related to area of specialization. For example, among practicing civil engineers, only 39% of design civil engineers were left brained, compared to 74% of construction civil engineers. Morton (2003d) suggested that individuals in primarily “top-down” professions working at structural levels that are subdivisible, such as microbiologists, biochemists, and particle physicists, were more left brained. In contrast, those in more “bottom-up” macroscopic or gestalt-oriented professions such as architecture, civil engineering design, and astronomy, tended to be more right brained. Thus, as it may be seen, hemisity appears to play a profound role in career development.

An explanation has been proposed to account for the sorting of hemisity in higher education and career selection (Morton, 2003d). That is, sorting occurred as the result of RPs and LPs doing what they liked best. Topics at which each excelled relative to the other resulted in one hemisity subclass doing well or poorly compared to the other. Rewards from success, difficulty, or failure shaped individual opinion of the liking or dislike of specific topics. This led to the selection of topics bringing personal success and to the avoidance of those bringing failure. Thus, in general, it appears that one ends up being an architect or microbiologist simply by doing what one enjoys most.

Although both the Best Hand Test (Morton, 2003b) and the Polarity Questionnaire (Morton, 2002) were used in the above population studies, the viability of using the more easily administered Polarity Questionnaire alone to determine the hemisity of large groups was considered by comparing its outcomes here with those of the Best Hand Test alone (Morton, 2003b). For a high school population (*n* = 703), the outcomes of the two methods differed in only 5.6% of cases. Further, the Polarity Questionnaire was able to assess the hemisity of the 10.4% individuals whose Best Hand Test results were indeterminate. This supported the idea that, not only are the two measures complimentary, but also that perhaps future studies using the Polarity Questionnaire alone, or in combination with one or more of the other calibrated hemisity questionnaires might be acceptably accurate for the estimation of hemisity of large English speaking populations. However, the extreme outcome sensitivity to wording of Polarity Questionnaire statements (Morton, 2002, 2003c) suggests that great care must be taken in its translation into other languages and cultures. In contrast, biophysical hemisity methods, such as the Best Hand Test, while much more demanding to assess, appear to be language and culture independent.

Because the grading of the Best Hand Test, a research instrument, is complex, technical, and time consuming, it is not practical for use in general hemisity studies. As indicated above, similar results are easily obtained by the Polarity Questionnaire. Further, it has been shown that combined use of the Polarity Questionnaire with the three other rapid binary hemisity questionnaires that have been developed: the Asymmetry Questionnaire (Morton, 2003c), the Binary Questionnaire (Morton, 2012), and the Hemisity Questionnaire (Morton, 2012), enhances the 80% certainty of the hemisity subtype result of a single questionnaire to about 95% for combined use (Table 2.) Each questionnaire takes only a few minutes to administer and grade.

## Conclusions

Six useful conclusions are among many that can be derived from this review of hemisity: (1) Research now supports the view that the existence of hemisity is inevitable, due to the unilateral nature of a structural element of the executive system. (2) Quantitative methods have been developed to make it possible to assess any person in terms of their probable right or left brain orientation. (3) A primary standard has been discovered that enables the absolute hemisity of an individual to be determined, based upon anatomical landmarks within the brain. (4) A number of the many “either-or” traits that separate the cognitive and behavioral styles of RPs and LPs have been identified, most of which as yet have no known ties to brain asymmetry. (5) Methods now exist which can determine the average hemisity of groups with considerable sensitivity. (6) The recognition of the quantifiable existence of hemisity as a second dyadic personal identifier after sex can bring new clarity to human behavior.

The neuroanatomical differences between left- and right-brain oriented individuals raise the question of how these features develop. Correlating parent and offspring hemisity types might provide first insights into the development of this phenomenon. However, extensive genetic research will most likely be necessary to fully unravel the development and implications of hemisity.

### Conflict of interest statement

The author declares that the research was conducted in the absence of any commercial or financial relationships that could be construed as a potential conflict of interest.
